# Directions for the International Society for Research on Internet Interventions (ISRII)

**DOI:** 10.2196/jmir.8.3.e23

**Published:** 2006-09-29

**Authors:** Lee M Ritterband, Gerhard Andersson, Helen M Christensen, Per Carlbring, Pim Cuijpers

**Affiliations:** ^4^Vrije Universiteit of AmsterdamAmsterdamThe Netherlands; ^3^Australian National UniversityCanberraAustralia; ^2^Linköping UniversityLinköpingSweden; ^1^University of Virginia Health SystemCharlottesvilleVAUSA

**Keywords:** ISRII, Internet interventions, Online treatment

## Abstract

In 2004, the International Society for Research on Internet Interventions (ISRII) was formed to encourage eHealth researchers to collaborate in their efforts to further the science behind developing, testing, and disseminating Web-based treatment programs. The group held its second meeting (April 2006) to clarify the Society’s direction and identify key issues that need addressing in the field. These issues are identified and examined in the current paper. Given the success of using the Internet to treat a range of medical and mental health problems, and the growing need for better dissemination of health care, Internet interventions will almost certainly play a prominent role in global health. ISRII plans to provide the necessary venue to ensure the science driving this field is strong, enabling researchers to conduct the highest quality research and permitting meaningful conclusions from completed studies.

## Introduction

The growth in Internet interventions for mental health and behavioral health programs has increased rapidly in the past decade (see [1]). Internet interventions are treatments, typically behaviorally based, that are operationalized and transformed for delivery via the Internet. Usually, they are highly structured; self-guided or partly self-guided; based on effective face-to-face interventions; personalized to the user; interactive; enhanced by graphics, animations, audio, and video; and tailored to provide follow-up and feedback [2]. As of early 2006, there were over 25 randomized controlled trials of Internet-based mental health interventions (see reviews [3-6]), and, based on the 2006 meeting of the International Society for Research on Internet Interventions (ISRII) at the Karolinska Institute in Stockholm, Sweden, at least 10 additional Internet intervention trials are nearing completion or are being analyzed for publication (see www.isrii.org for a listing of presentations at the 2006 meeting).

While the *feasibility* (can the intervention be delivered) of Internet health interventions in general has been well validated, *efficacy* (is the intervention successful when delivered under controlled conditions [7]) of Internet applications has now also been established for a number of health problems, including anxiety (eg, panic [8-10], post-traumatic stress disorder [11-13], social anxiety disorder [14,15]), depression [16-18], eating disorders (eg, weight loss [19,20], binge eating and bulimia [21]), body image [22,23], insomnia [24], and more general medical areas such as headache [25,26], back pain [27], diabetes management [28], encopresis [29], tinnitus [30-32], and smoking cessation [33]. True *effectiveness* (is the intervention successful in actual clinical practice [7]) and cost-effectiveness trials are underway.

Clearly, there is an appreciation for this new form of treatment and its unique ability to be widely disseminated as millions of dollars have already been allocated by the National Institutes of Health in the United States and other government agencies and various industries around the world. The recommendation of computer-based interventions such as Fear Fighter [34,35] (for anxiety) and Beating the Blues [36,37] (for depression) by the United Kingdom’s National Institute for Health and Clinical Excellence (NICE) introduces a new level of acceptance by government and medical insurers of the feasibility and value of such interventions [38]. This acceptance is an essential step in establishing this mode of treatment delivery (via computers and the Internet). This, along with investigating how Internet interventions compare with more traditional forms of treatment delivery (eg, bibliotherapy, individual and group face-to-face, telephone), will be important in clearly establishing Internet interventions as a viable and effective form of treatment, as well as demonstrating their ability to change behaviors and improve symptoms, cost-effectiveness, scalability, and acceptance in the community.

ISRII is an organization of researchers from around the world whose focus is on the development and testing of various Web-based health treatment programs. The primary aim of the Society is to promote the exchange of ideas and experiences among researchers involved in intervention research using the Internet. Among the many interests shared by the members are cognitive behavioral interventions using the Internet, technical solutions in Web applications, Web-based questionnaire assessments, and computer applications in clinical psychology and in psychiatry more broadly. At the April 2006 meeting, the organization confirmed its aims and identified a number of key issues for the future.

## Key Aims and Directions

### To continue to conduct the highest quality research and further establish the science of Internet interventions

The primary goal of ISRII is to continue high-quality research to determine the efficacy and effectiveness of health applications on the Internet, which is vital to the future of this mode of treatment. In particular, the Society aims to establish an evidence base for the usefulness of Internet applications across a range of disorders and diseases. This objective represented a major drive for the first ISRII conference in 2004. At these meetings, experts convene to critically review and improve the quality of intervention research undertaken by Society members. By ensuring that programs are empirically validated, reliable, and appropriately generalizable, ISRII distinguishes them from other industry, commercial, or otherwise nonempirically based Web programs.

### To facilitate collaboration among Internet intervention researchers

Internet applications are potentially global, and collaboration in research and dissemination is likely to improve the quality and scope of the research, reduce disease burden, and improve outcomes. In particular, there is recognition among the Society researchers of the importance of working strategically to develop and evaluate Internet applications. Scalable, interactive applications are costly and time-consuming to produce, especially if they are to be empirically validated. There is significant potential in strategically developing new websites to reduce duplication and to avoid “dead ends.”

### To better understand how behavior change and symptom improvement are produced through the use of Internet interventions

The chief goal of any Internet intervention is to produce cognitive and behavior change that leads to symptom improvement. Examining and testing this process using theories and models of behavior change is critical to furthering the understanding of how Internet interventions, and even treatments in general, work. Models specific to Internet interventions are needed as there are obvious differences in treatment delivery from traditional interventions. Evaluating the Internet as the platform for delivering treatments has some significant advantages over testing more traditional approaches. An advantage of conducting randomized controlled trials through the Internet is the ease of obtaining large sample sizes (no geographical limitations), making it possible to better examine mediators and moderators of treatment. In addition, deconstructing treatments is perfectly suited to Internet intervention research in that the programs are already operationalized and can be readily compartmentalized and studied separately. This may allow for a much better examination of the nonspecific variables of treatment than has been done in the past.

### To implement and disseminate Internet applications to anyone, anywhere

A strength of the Society lies in the expertise of its members in delivering Internet applications at a community or population level. Models for the dissemination and implementation of scalable interventions are needed. A special function of the Society will be to organize translation of applications into languages other than the original development language in order to permit broader dissemination.

### To develop an understanding of who will use Internet interventions and how to encourage adherence

Determining who wants to use and who is likely to use Internet interventions are important issues to consider for purposes of dissemination. Examining characteristics of Internet intervention users will help not only improve the tailored nature of these programs, but also help better predict outcomes. Poor adherence is a significant issue for most health interventions, including Internet interventions. The World Health Report of 2002 declared that adherence was the primary determinant of the effectiveness of treatment [39]. This issue of adherence is a critical one for Internet intervention researchers and a key area of focus for the Society. Developing ways to reduce attrition, improve adherence, and maintain compliance is a major objective for members of the Society.

### To use Internet research applications to collect minimum data sets

The inclusion of standard measures, such as the EuroQol (EQ-5D) [40] or the SF-12 [41], will allow comparisons across health care systems (eg, Internet communities, clinical groups, and formal health care services), across health problem samples (eg, applications for depression in comparison to diabetes), and within Internet applications (eg, pre- to post-symptom change due to a program for panic).

### To examine and validate current tests and measures for Internet delivery

Before well-validated, paper-and-pencil measures should be delivered online, a process of validating these tests in this new mode should be made. While not a major area of focus for this Society, it is an important related issue which most Internet intervention researchers manage.

### To examine and test the validity of a range of new online tests and assessments

Validating new online tests and assessments will lead to a library of useful and valid Internet assessments. For example, creating and testing shortened versions of various psychological scales would be relatively easily achieved by using online surveys to validate the items compared to longer versions. There are multiple reasons to develop and validate briefer tests, including the recognition that the Internet is used in short bursts. Data collection can be rapid on the Internet, especially on open-access sites. The Internet also offers the possibility to considerably reduce the length of questionnaires while maintaining reliability. These “adaptive testing techniques” have been shown to reduce the number of necessary items to almost a quarter of that needed in paper versions of questionnaires [42] without any loss of accuracy. This area extends to other psychological tests, such as measures of information processing and neuropsychological testing via the Internet.

### To provide a forum to examine models of commercialization

Given the time and resources invested in developing and testing Internet interventions, making these programs available to the public is often an important goal. Examining models of commercialization and dissemination to determine how best to make these programs available is critical. Given the research focus of the Society, these models might typically incorporate commercialization that allows continued research and evaluation. A range of business models are available to develop research prototypes into fully scaled applications. There may be joint business opportunities for groups of researchers. Discussions should lead to an understanding of how health structures and health system remuneration within countries influence methods of commercialization. Many researchers are interested in learning how their applications might be sustained when research funding ends. While commercializing is a potentially important area of focus, dissemination, however it may occur, is the goal.

### To establish guidelines and parameters for the use of current and future interventions

Given the growth and consumer interest in Internet interventions, it is essential to establish and implement guidelines to identify and tag empirically validated, reliable, and effective applications. At a minimum, statements about minimal guidelines for quality and effectiveness are necessary. The Society also seeks to develop a classification scheme to differentiate intervention types (eg, information-only, interactive with information and decision support, interactive with additional human support) in order to enhance comparisons across applications, as well as improve consumer accessibility and understanding. This would likely also enrich future meta-analyses.

These 10 key issues constitute the main aims and directions of the ISRII. Given the wealth of experience, research, and dedication to the science of this discipline, ISRII expects to make significant and substantive contributions to the field of Internet interventions. The possibility of impacting countless lives with the ability to disseminate interventions anywhere in the world makes the mission of this Society a critical and rewarding endeavour.

## Abbreviations

ISRII: International Society for Research on Internet Interventions
NICE: National Institute for Health and Clinical Excellence
